# The importance of considering the possibility of ocular sporotrichosis in areas with high incidence rates of sporotrichosis

**DOI:** 10.5935/0004-2749.20230062

**Published:** 2023

**Authors:** Mariana Nadais Aidar, Barbara Millani Rebeschini, Carolina Tiburcio Salgado Silveira da Mata, Thaís Cândida Borges, Maria Emília Xavier dos Santos Araújo

**Affiliations:** 1 Ophthalmology Service, Hospital do Servidor Público Estadual de São Paulo, São Paulo, SP, Brazil.

**Keywords:** Eye infections, fungal, Conjunctivitis, Sporotrichosis/drug therapy, Sporothrix/isolation & purification, Itraconazole/therapeutic use, Infecções oculares fúngicas, Conjuntivite, Esporotricose/tratamento farmacológico, Sporothrix/isolamento e purificação, Itraconazol/uso terapêutico

## Abstract

Ocular sporotrichosis involving adnexa can present in 4 types: granulomatous conjunctivitis, dacryocystitis, Parinaud oculoglandular syndrome, and bulbar conjunctivitis. The incidence of ocular sporotrichosis has increased in regions with high incidence rates of sporotrichosis. We present a series of three cases of ocular involvement by the fungus *Sporothrix* species, including its manifestations, approaches, and relevance in areas where sporotrichosis has considerable incidence rates.

## Abstract (pt)

A esporotricose ocular envolvendo anexos pode se apresentar de quatro formas: conjuntivite granulomatosa, dacriocistite, Síndrome Oculoglandular de Parinaud e conjuntivite bulbar. A esporotricose ocular, apesar de incomum, tem aumentado em regiões com alta incidência de esporotricose. Apresentamos uma série de três casos de envolvimento ocular pelo fungo *Sporothrix sp*.: suas manifestações, abordagem e sua relevância em áreas com alta incidência de esporotricose.

## INTRODUCTION

Parinaud oculoglandular syndrome (POS) is a rare clinical condition characterized by granulomatous conjunctivitis associated with preauricular or submandi­bular lymphadenopathy ipsilaterally^(1)^.

The main etiological agent of POS is *Bartonella henselae*^(2,3)^, although other organisms have also been described^(1)^. The sporotrichosis-causing fungus is described as the third leading cause of POS, after cat-scratch disease and tularemia.

Besides POS, ocular sporotrichosis involving adnexa can also present with granulomatous conjunctivitis, dacryocystitis, and bulbar conjunctivitis^(4)^. The incidence of ocular sporotrichosis has been reported to increase in regions with considerable incidence rates of sporotrichosis^(5)^.

We present a case series of ocular involvement caused by a fungus of the *Sporothrix* species, including its manifestations and management, which is relevant in areas with high incidence rates of sporotrichosis.

## CASE REPORTS

### Case 1

**Figure 1:**
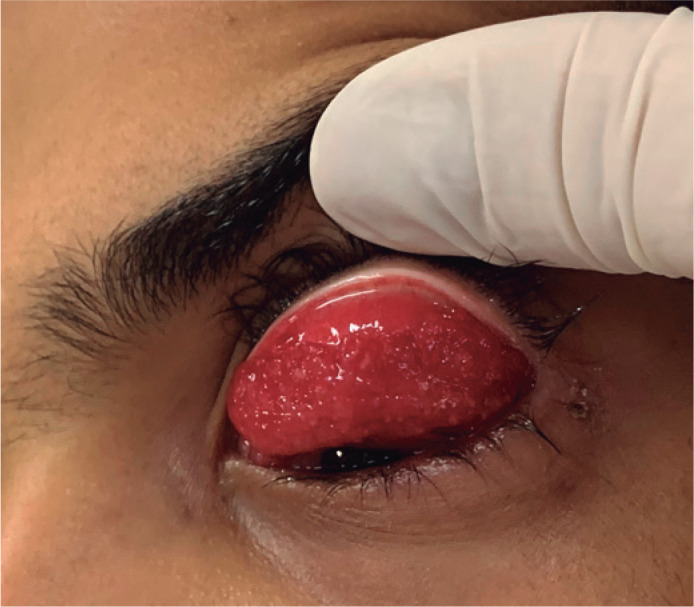
Follicular reaction and granulomas in the upper tarsal conjunctiva.

**Figure 2:**
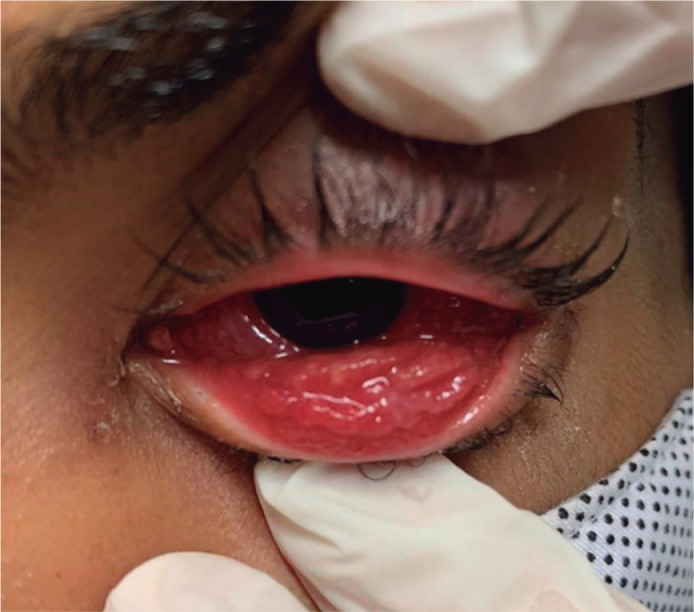
Follicular reaction and granulomas in the lower tarsal conjunctiva.

**Figure 3:**
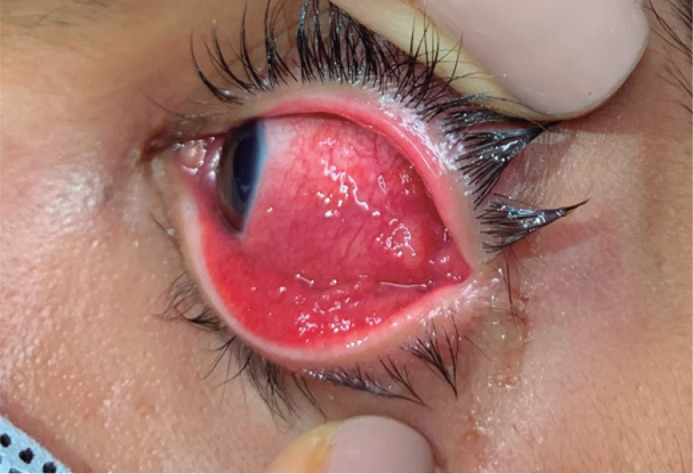
Conjunctival granuloma in the outer corner of the left eye, where biopsy was performed.

**Figure 4:**
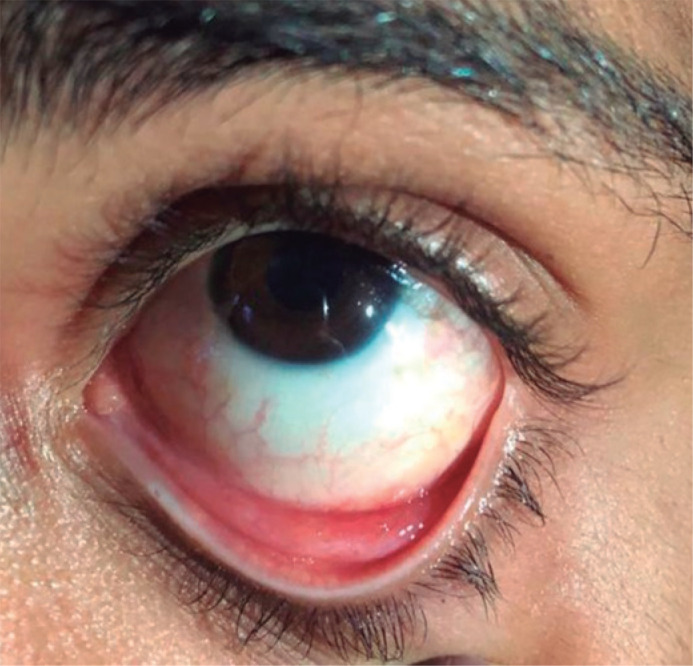
Appearance of the eye during external eye examination, showing total resolution after a 2-month treatment with itraconazole.

A 17-year-old male patient presented with a 2-week history of symptoms of pain and hyperemia in the left eye. He mentioned contact with various stray cats in the previous weeks. Clinical examination revealed follicles and granulomas in the upper (Figure 1) and lower tarsal conjunctiva (Figure 2) and ipsilateral submandibular and preauricular lymphadenopathy. No other abnormal findings were obtained. The patient was positive for IgM toxoplasmosis and negative for cat-scratch disease. Treatment for toxoplasmosis was administered before the patient’s visit to our service, and treatment for cat-scratch disease (doxycycline 100 mg twice daily for 2 weeks) was prescribed, but no improvement was observed. Cultures of specimens from scrapings and conjunctival secretions and a histopathological study of the conjunctival granuloma in the outer corner of the left eye were performed (Figure 3), which showed granulomas but not the causative agent. No fungal growth was observed. Itraconazole 200 mg/day was administered, with total regression in 2 months of treatment (Figure 4).

### Case 2

A 6-year-old boy was brought to our clinic by his mother for evaluation due to ocular hyperemia in the right eye, which had persisted for 3 weeks. The mother mentioned previous treatment with topical tobramycin for 1 week and oral erythromycin for 2 weeks, with no improvement. She described a history of feline sporotrichosis in her domestic animal, with a positive culture result of the lesion sample. Clinical examination of the child revealed follicles in the upper and lower tarsal conjunctiva, temporal conjunctival hyperemia, and diffuse granulomas in the bulbar conjunctiva. Ipsilateral cervical lymphadenopathy was detected. Other findings were unremarkable. The patient’s clinical presentation and epidemiological findings suggested POS due to sporotrichosis. Therefore, treatment was initiated with itraconazole 100 mg twice daily for 2 months, which achieved total regression.

### Case 3

An 18-year-old female patient complained about a growing nodule in her right lower eyelid associated with eye discomfort, which started 15 days before. She was previously diagnosed as having a chalazion and treated with corticosteroid, antibiotic ointment, oral doxycycline, and warm compresses, with no improvement. She mentioned that her cat had dermatological lesions diagnosed as sporotrichosis through a skin biopsy. Her clinical examination revealed multiple slightly hyperemic granulomatous lesions on the upper and lower tarsal conjunctiva of the right eye, which was associated with a follicular reaction. At the time of examination, no palpable lymph nodes were found. Other findings were unremarkable. As the clinical presentation and epidemiological findings suggested ocular sporotrichosis, itraconazole was prescribed at 200 mg daily for 30 days, which improved the patient’s condition.

## DISCUSSION

Fungal infections are generally neglected, and inadequate surveillance leads to their emergence, as observed in zoonotic sporotrichosis. Brazil experienced a geographic expansion of sporotrichosis during 1998-2017, and the incidence of zoonotic sporotrichosis (disease transmitted from animals to humans) has increased considerably^(5)^. The southeast region had the highest incidence rates of human and animal cases^(6)^. Some factors can be cited as causes of this increase, such as socioeconomic and environmental difficulties, urban overcrowding, and poor basic sanitation^(5)^.

Even with the spread of the disease to other states in Brazil, compulsory notification in human cases only occurred in the states of Rio de Janeiro, Pernambuco, and Paraíba and in the municipalities of Guarulhos (SP) and Salvador (BA)^(7)^. Since 2011, as a result of the frequent increase in the number of sporotrichosis cases, the São Paulo municipality has been conducting widespread surveillance of sporotrichosis cases. However, the municipal decree that defined notification of suspected cases of sporotrichosis in animals and humans as mandatory in São Paulo municipality was published only in 2020^(5)^.

Recently, Ribeiro et al. reported a series of 10 cases of POS due to contact with infected domestic cats in the São Paulo metropolitan region, emphasizing the importance of considering ocular sporotrichosis as a differential diagnosis^(8)^. In addition, Yamagata et al.^(9)^ described ocular sporotrichosis as an often-misdiagnosed cause of granulomatous conjunctivitis in endemic areas.

Ocular sporotrichosis can develop hematogenously or by the involvement of ocular adnexa due to self-ino­culation or trauma^(10)^. Sporotrichosis ocular involvement has rarely been described in immunocompetent patients or in individuals without previous ocular trauma^(8)^. Our three patients had no history of trauma or relevant past medical history (all of them were immunocompetent), which leads us to the hypothesis of contamination by self-inoculation due to contact with contaminated felines.

When it comes to ocular sporotrichosis involving adnexa, some possibilities of manifestation include granulomatous conjunctivitis, POS, dacryocystitis, and bulbar conjunctivitis^(4)^. In granulomatous conjunctivitis, clustered nodules appear with a smooth and shiny surface surrounding the tarsal and/or bulbar conjunctiva, associated with conjunctival hyperemia^(9)^.

At the onset of the disease, granulomas can be confused with hordeolum and chalazion, which leads to delay of the correct diagnosis, as in cases 2 and 3^(11)^. Ocular sporotrichosis is an important differential diagnosis in lesions that do not respond to standard treatments and should be considered especially in areas with high incidence of sporotrichosis.

The gold standard diagnostic method for ocular sporotrichosis is the collection of conjunctival discharge sample with a sterile swab and culturing the material for fungi or by granuloma biopsy^(8)^. However, in our three cases, clinical and epidemiological history-presumptive diagnosis was made. *Itraconazole* at a dose of 100-200 mg daily is effective and well tolerated and has largely replaced spectrum selective kinase inhibitor and amphotericin B owing to its 90-100% efficacy rates for cutaneous and extracutaneous sporotrichosis^(12)^. Our patients were treated with 200-mg itraconazole daily and showed great therapeutic response with total regression.

As only few reports have described ocular sporotrichosis and sporotrichosis has not been described as the first diagnostic hypothesis in cases of POS, diagnosis and treatment are delayed. Owing to the increasing incidence of sporotrichosis in certain regions, the incidence of ocular sporotrichosis has also increased. Therefore, in these areas, including sporotrichosis among the first diagnostic hypotheses is relevant in cases of granulomatous conjunctivitis, POS, dacryocystitis, or bulbar conjunctivitis.

We emphasize the importance of ophthalmologists being familiar with the differential diagnosis in cases of granulomatous conjunctivitis that does not improve with conventional treatment, especially in areas with increased incidence of sporotrichosis, and encourage compulsory notification of animal and human cases of ocular sporotrichosis.
